# Intermittent Hypoxia Interferes with Autocrine Effects of GABA on Insulin Secretion in Postnatal Rodents—Implications for Pediatric Obstructive Sleep Apnea

**DOI:** 10.3390/children9091305

**Published:** 2022-08-28

**Authors:** Eung-Kwon Pae, Man-Kyo Chung, Ronald M. Harper

**Affiliations:** 1 School of Dentistry, University of Maryland, Baltimore, MD 21201, USA; 2 Department of Neural and Pain Sciences, School of Dentistry, Center to Advance Chronic Pain Research, University of Maryland, Baltimore, MD 21201, USA; 3 Department of Neurobiology, University of California at Los Angeles, Los Angeles, CA 90095, USA

**Keywords:** chloride, GABA_A_ receptors, insulin, intermittent hypoxia, pediatric obstructive sleep apnea

## Abstract

Gamma-amino butyric acid (GABA) is well known to help elevate pancreatic β cell vitality and insulin levels in blood. GABA works via a coupling with GABA receptors; thus, the concentration of GABA_A_ receptors on the plasma membrane of β cells appears to be critical for insulin regulation. Various medical conditions, such as pediatric and adult obstructive sleep apnea (OSA), show high levels of Type 2 diabetes; such patients also are exposed to intermittent hypoxia (IH), which modifies the GABA levels. To evaluate the potential therapeutic roles of GABA for diabetic patients with OSA, we studied the interactions of IH with GABA and GABA_A_ receptors in young rats. Using rat pups and primary pancreatic islets, we evaluated the roles of GABA in insulin secretion. We show that GABA effectively increased the insulin secretion of pancreatic islets under normal ambient oxygen levels, as well as in culture medium with a glucose level of 2 mM. GABA also increased islet insulin secretion conditioned under IH in a 16 mM glucose medium. When islets were IH-treated, insulin secretion decreased due to lower intracellular chloride levels in accordance with the increased KCC2 levels. The results show that IH challenges down-regulate the GABA_A_ receptor levels in pancreatic islets, which decreases GABA–GABA_A_ receptor coupling action, as well as membrane depolarization for insulin secretion. The findings have the potential to suggest novel interventions for insulin regulation during IH of disordered breathing, including OSA.

## 1. Introduction

Preterm birth is accompanied by low birth weight and apnea of prematurity. Rapid weight gain after birth can be associated with postnatal glycemia, insulinemia, higher blood pressure and an increased adiposity, which predisposes the infant to an increased risk of obesity and cardiometabolic outcomes at a later age [1]. In addition, in one study, 40% of pediatric obstructive sleep apnea (OSA) patients showed Type 2 diabetes (T2D) [2]. We suggest that gamma-amino butyric acid (GABA) could assist in such conditions in children.

GABA, a common inhibitory neurotransmitter in the central nervous system, is also an extracellular signaling molecule released from insulin-secreting pancreatic β cells [3,4]. GABA modulates insulin exocytosis and glucose-stimulated insulin secretion. Previous studies have revealed that the excitability of the pancreatic β cell membrane increases as chloride ion permeability increases [5,6,7]. A chloride ion-channel GABA_A_ receptor is a pentameric transmembrane protein complex with a chloride ion-permeable pore in the center. The channel is normally closed, yet it opens as conformational changes occur while GABA binds to the GABA_A_ receptor, and then moves chloride ions into the cell. Earlier studies showed that GABA_A_ receptors that couple with GABA molecules open these channels in β cells [8]. Thus, GABA plays an important role as an autocrine and/or paracrine modulator of insulin release in pancreatic islets [8,9]. When GABA reaches saturation levels, the channels are desensitized and cease working [8]. GABA signaling processes appear to be GABA_A_ receptor quantity-sensitive.

The administration of GABA significantly increases the circulating insulin levels in blood under either fasting or satiated conditions after single or repeated doses [10]. Because GABA is rapidly absorbed within 1 h with a relatively short half-life of 5 h in rodents, administration is considered a supplementary regimen for assisting Type 2 diabetic (T2D) conditions [11,12]. However, whether GABA can be employed as a therapeutic regimen for T2D symptoms in patients with OSA, a condition in which the body is exposed to repetitive intermittent hypoxia (IH) exposure and is frequently accompanied by high levels of T2D, is uncertain. Maintaining insulin levels via GABA effects is more challenging for patients with OSA, because GABA influences insulin secretion under IH conditions induced by OSA, which could be unsafe; GABA also plays sedative roles that may further compromise breathing. Furthermore, a recent study on large samples of adult patients reported a higher odds ratio of up to 24% of sleep disturbance amongst patients taking non-metformin in comparison with those without antidiabetics [13], raising concerns of GABA’s effects on state transitions to sleep.

Recently, we showed that low blood insulin levels due to IH exposure are a consequence of decreased levels of chloride in β-cells after a challenge. The concentration of [Cl^−^]_i_ is modulated by the amount of γ-aminobutyric acid receptor subtype A [7,8], as well as the ratio between chloride importer Na^+^-K^+^-Cl^−^ cotransporters and chloride exporter K^+^-Cl^−^ cotransporters (KCC2) in the β cell membrane [6,14]. A significant decline in GABA_A_ receptors, as previously observed in neuronal cells [15,16], results in low intracellular chloride levels. If the [Cl^−^]_i_ in β cells that is dependent on GABA_A_ receptors and K^+^-Cl^−^ cotransporters is disrupted, membrane depolarization would not occur [5,6,7,8]. Thus, we propose a hypothesis rationalizing a close interaction between IH insult and down-regulated insulin secretion levels via a lack of GABA–GABA_A_ receptor coupling actions.

We hypothesized that IH challenges to primary islets of rat pups reduced the down-regulation of GABA_A_ receptors in islet β cells. Such down-regulation of the GABA_A_ receptor levels results in decreased insulin secretion due to reduced intracellular chloride levels. We aimed to demonstrate this set of outcomes in rat pups to understand the potential relationship between OSA and T2D. We also aimed to raise the possibility that the use of GABA alone may serve as a complementary regimen for T2D in patients with OSA.

## 2. Materials and Methods

### 2.1. Intermittent Hypoxia Exposure to Islets

Primary pancreatic islets harvested from male rat pups were used to examine (1) if exogenous GABA alters intracellular calcium ion levels for insulin secretion and (2) IH effects on insulin secretion and signaling molecules along the pathways associated with insulin production and secretion.

**Figure 1:**
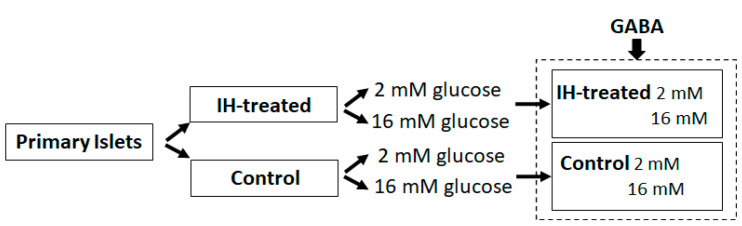
Summary of in vitro studies. Bicuculin or tolbutamide was applied instead of GABA as needed.

The isolated islets obtained from several pups were treated in a hypoxia chamber (Billups-Rothenberg Inc., San Diego, CA, USA), and the chamber air was intermittently altered between room air (ambient oxygen level approximately 21%) and a hypoxic gas mixture (1% O_2_, 5% CO_2_ and 94% N_2_) every 10 min for ten cycles (3 h in total) as described previously [17]. For the stimulation of insulin secretion in an optimized condition, IH-pretreated islets were incubated for 12 h for stabilization and then compared with control islets in two distinct glucose media. Two different glucose levels (2 mM vs. 16 mM) were used for the fasting-like and postprandial-like conditions in children with glycemia and insulinemia. The study design is summarized below (See Figure 1):

### 2.2. Intermittent Hypoxia Exposure to Animals

To investigate IH influences on GABA_A_ receptors, we used 3-week-old animals. Approximately 2–3 h after parturition, pups and their mothers of the experimental group (*n* = 5) were maintained for 1 h within a chamber in which the oxygen level alternated between room air (21% O_2_) and hypoxic conditions (10% O_2_) every 4 min, mimicking apnea of prematurity in preterm human infants or OSA in children, as described previously elsewhere [17]. Only male pups were used to avoid potential sex-biased outcomes [18].

Randomly designated offspring and mothers (*n* = 5) in the control group were exposed to room air (RA). The litter size was matched between the control and IH groups to standardize the weight of each pup. After the IH and RA treatments, all pups were housed in regular husbandry in RA for 3 weeks. All animals were euthanized 3 weeks after IH and RA treatment.

### 2.3. Ethical Approval

The experimental protocol was approved by the institutional Animal Review Committee (ARC #2007-008-02, UCLA) and IACUC (#D121101, University of Maryland, Baltimore). All experimental methods were performed in accordance with the relevant guidelines and regulations constituted by the institutions.

### 2.4. Isolation of Islets and Subcellular Protein Fractionation

Details of the tissue procurement methods have been described previously [17]. In brief, the animals were sacrificed in a CO_2_ chamber and central blood was drawn from the heart. The serum was separated from centrifuged blood and stored for ELISA assays. For the isolation of islets, ice-cold collagenase solution was injected through the common bile duct. The removed pancreas was digested in collagenase at 37 °C for 8 min and washed twice with G-solution to delay the digestive process. The tissue was centrifuged and the pellet was re-suspended and then separated by gradient centrifugation. After the addition of 10% fetal bovine serum and 1% penicillin–streptomycin mixture to the supernatant, the islets in the media were maintained at 37 °C and 5% CO_2_ for 4 h.

### 2.5. Ratiometric Calcium Imaging

Ratiometric Ca^2+^ imaging analysis was performed as described previously in detail [19]. Dissociated islet cells were loaded with Fura2-AM (Molecular Probes) for 40 min at 37 °C in a calcium-imaging buffer (CIB) containing 130 mM NaCl, 3 mM KCl, 0.5 mM MgCl_2_, 0.9 mM CaCl_2_, 10 mM HEPES, 10 mM sucrose, 1.2 mM NaHCO_3_ pH 7.45 and 320 mOsm (adjusted with mannitol). Ratiometric Ca^2+^ imaging was performed using an inverted fluorescence microscope (Nikon Instruments, Melville, NY, USA) with an excitation filter changer (Sutter Instruments, Novato, CA, USA) and a cooled CCD camera (Nikon Instruments) at room temperature. After a 15-min incubation period for de-esterification, the coverslips containing the islet cells were placed in a recording chamber, which was continuously perfused with CIB with or without drugs. Dual images (510 nm emission) were collected every 2 s using NIS Elements (Nikon Instruments). The Fura response was defined as the ratio of emissions measured during excitation at 340 and 380 nm, and the relative Fura response was defined as the ratio with respect to the baseline Fura ratio. To assess the amplitude of changes in the relative Fura ratio (Δ Response) in each GABA administration, the baseline Fura ratios were calculated prior to the 1st, 2nd and 3rd applications, and the percent increase in the relative Fura ratio was calculated.

### 2.6. ELISA

Assays for ELISA to quantify insulin from blood or cultured media were performed using an ELISA Kit (EMD Millipore Corp, Burlington, MA, USA) in accordance with the protocol provided by the manufacturer, as previously described elsewhere [17].

### 2.7. Western Blot Assays

Whole-cell lysates were used for Western blot assays as previously outlined [17]. Shortly, the lysates (50 µg/sample) were separated using SDS-PAGE (**s**odium **d**odecyl **s**ulfate–**p**oly**a**crylamide **g**el **e**lectrophoresis) and transferred onto a **p**oly**v**inylidene **d**i**f**luoride (PVDF) membrane. For primary antibodies, GABA_A_ receptors α1, α2, α3 (R&D system, Minneapolis, MN, USA) and KCC2 (Santa Cruz Biotech, Dallas, TX, USA) were used. For visualization, a horseradish peroxidase-conjugated secondary antibody was used. Each protein band was visualized in Multi Gauge v3.0 (Fujifilm, Hanover Park, IL, USA) in reference to β-actin. Quantification and statistical tests on the density of protein bands for comparison purposes were not performed.

### 2.8. Colorimetric Assay for Chloride Quantification

The chloride content was measured using Colorimetric Assay Kits (BioVision, Inc., Milpitas, CA, USA) as detailed previously [14]. As mercuric chlorides liberated a color-quantifiable complex, iron-TPTZ (2,4,6-Tripyridyl-S-triazine), the absorbance was measured at 620 nm with a spectrophotometer. Note that these values are not direct measures of chloride ions in the cell cytosol.

### 2.9. RNA Extraction and Quantitative Real-Time RT-PCR

The total RNA was extracted from the harvested islets using an RNeasy Mini Kit (QIAGEN Sciences, Germantown, MD, USA), as previously described elsewhere [17]. The gene-specific primers are displayed in Table 1. Each sample was obtained from 3 different animals. C_T_, i.e., the threshold cycle value, was measured 3 times and each was normalized to a β-actin control. C_T_ measurements were used for group comparisons.

### 2.10. Statistics

SPSS v. 21 and GraphPad Prism 9.3.1 were used for statistical inference tests. For comparisons between groups (for instance, Control vs. IH), Student’s t-tests and one-way ANOVA with Tukey’s multiple comparisons were performed. The means and standard errors were calculated; *p* < 0.05 was considered significant. The Fura ratios measured on each GABA application were compared using one-way repeated ANOVA.

## 3. Results

### 3.1. Intracellular Calcium Levels in Pancreatic β Cells Are Elevated by Exogenous GABA Challenge

**Figure 2:**
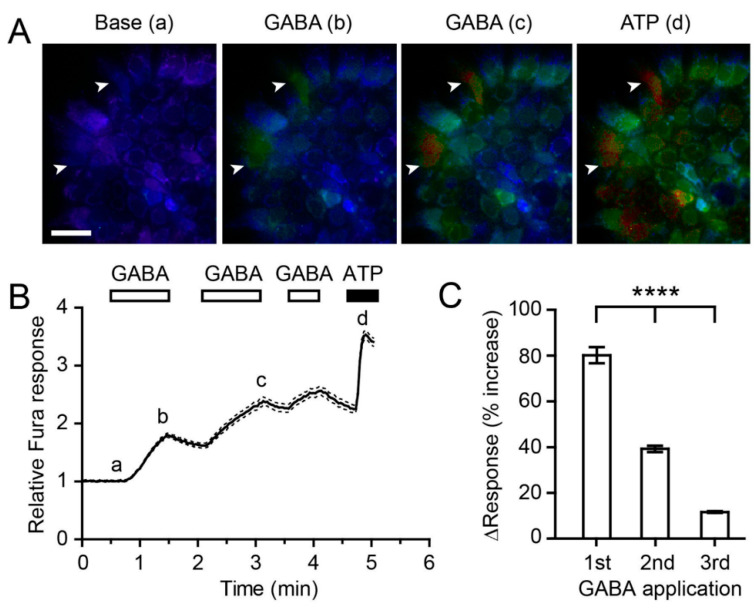
Exogenous gamma-aminobutyric acid (GABA) application increases the intracellular Ca^2+^ levels in pancreatic β-cells. (**A**) Representative pseudo-color images of the relative Fura ratios obtained at different time points, a to d, as indicated in (**B**); higher relative Fura responses are indicated by the red color. Arrowheads indicate two cells responding to GABA (time point b and c) and ATP (time point d). Scale bar = 50 μm. (**B**) Time-dependent changes in the normalized Fura ratio following the applications of GABA (500 µM) and ATP (100 µM). Mean (solid line) ± SEM (dashed lines). (**C**) Percent increase in response to GABA relative to the relative Fura ratio immediately prior to GABA application. **** *p* < 0.0001, one-way repeated-measure ANOVA, *n* = 74.

To evaluate whether β cell membranes were excitable by exogenous GABA application, we monitored changes in the intracellular Ca^2+^ levels in primary islet cells (*n* = 74) by ratiometric Ca^2+^ imaging using Fura2AM. Low intracellular Ca^2+^ levels were maintained under baseline conditions (Figure 2A,B; time point a). GABA (500 µM) bath perfusion increased the mean intracellular Ca^2+^ level by 1.8 ± 0.04 fold (*p* < 0.0001, Student’s paired *t*-test; *n* = 74) throughout the 1 min of application (Figure 2A,B; time point b). Upon washing out GABA, the Ca^2+^ levels decreased toward the baseline level, although the recovery was not complete during a 30 s washout. The second and third applications of GABA (indicated by the horizontal bars in Figure 2B) elevated the Ca^2+^ levels repeatedly (Figure 2A,B). The extent of net relative increases in Ca^2+^ relative to the previous time point was significantly smaller than the responses achieved by the previous applications (Figure 2C). At the end of the GABA application, ATP (100 µM) was perfused over the islets as a positive control. ATP application produced a robust increase in the Ca^2+^ levels, which validated the health of the islet cells, and GABA and ATP increases elevated the intracellular Ca^2+^ levels through distinct pathways. The results suggest that GABA elicits an increase in the intracellular calcium levels in pancreatic β cells, which depolarizes the cell membranes, likely leading to insulin secretion.

### 3.2. Responses of Primary Islets to IH Exposure to Different Glucose Levels

**Figure 3:**
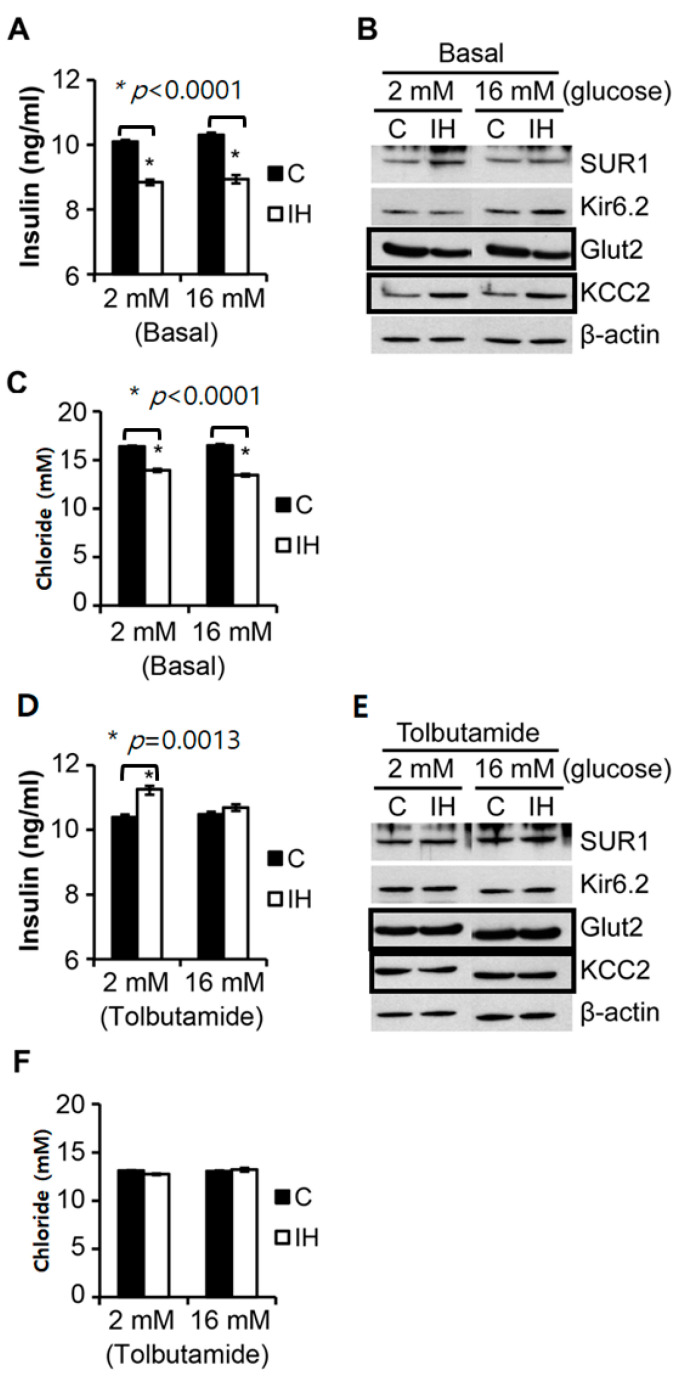
Comparisons of control vs. intermittent hypoxia (IH)-treated islets in the quantity of insulin secretion and the chloride levels measured from isolated primary islets at different glucose levels. IH treatment was performed 12 h prior to this set of assays. Insulin secretion quantified by Enzyme-linked immunosorbent assay (ELISA) (**A**), KCC2 levels compared using Western blots (**B**) and chloride content quantified by a colorimetric assay (**C**) following IH exposure in 2 mM and 16 mM glucose media. No influence from the different glucose levels in media was noted. Western blots for Glucose transporter 2 (Glut2) and KCC2 show a marked difference between the control and IH in both glucose media. Levels of sulfonylurea receptor 1 (Sur1) and ATP-sensitive K^+^ channel (Kir6.2) showed no marked changes. Addition of tolbutamide to 2 mM increased insulin secretion (**D**). Western blots showed minimal changes in response to tolbutamide (**E**) and the chloride levels showed no changes (**F**). Full-length blots are found in the Supplementary Information, Figure S1. * indicates significantly.

As shown in Figure 3, following 10 cycles of IH exposure for 3 h in total, the insulin secretion and levels of glucose transporter 2 (Glut2) were decreased in isolated primary islets; however, KCC2 expression increased notably, and the level of cellular chloride content decreased irrespective of the glucose levels. The glucose levels in each medium solution did not show noticeable effects with IH on insulin release, KCC2 expression, or the chloride levels (Figure 3A–C). In both 2 mM and 16 mM glucose media, a significant decrease was observed in insulin secretion (Figure 3A), along with a marked increase in KCC2 expression (Figure 3B) in Western blots after IH challenge. Glut2 expression showed a decrease after IH exposure in either 2 mM or 16 mM glucose media (Figure 3B). Sur1 and Kir6.2, subunits of the ATP-sensitive K^+^ channel, showed the minimum changes in Figure 2B. However, the intracellular chloride levels significantly decreased at *p* < 0.0001 in response to IH exposure (Figure 3C). When tolbutamide (10 mM), a potent potassium channel blocker binding to sulfonylurea receptors (Sur1), was added, the insulin levels in the media with 2 mM glucose only increased significantly.

We hypothesized that modulators of membrane hyperpolarization or depolarization for insulin secretion syncing with intracellular chloride levels may be mediated by GABA–GABA_A_ receptor coupling [8], as well as by the concentration of chloride cotransporters, such as KCC2 [5,6]. We observed that the intracellular chloride contents (i.e., arguably ‘chloride levels’ expressed in mM, where the measured numbers are the total amount of chloride (not chloride ion concentrations)) declined significantly (Figure 3C). When 10 mM of tolbutamide, an ATP-dependent potassium channel blocker, was added to the media, the quantity of insulin secreted to the medium with 2 mM glucose increased significantly (*p* = 0.0013). However, the intracellular chloride levels did not differ between control and IH in both media (Figure 3F).

### 3.3. IH Exposure for 1 h at Postnatal Day 1 (P0) Decreases Serum Insulin and Reduces GABA_A_ Receptor Levels in Pancreatic Islets

**Figure 4:**
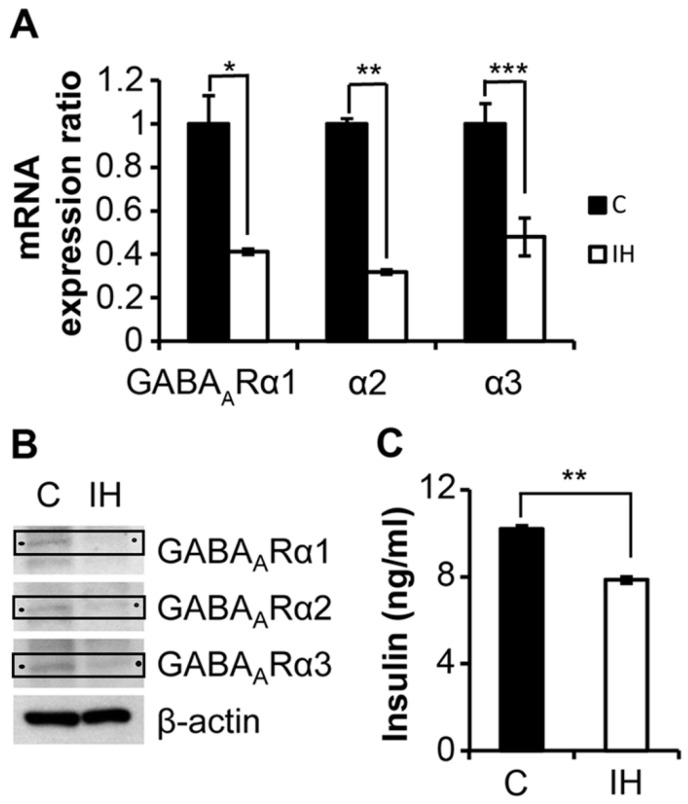
Comparisons between control and IH measurements on insulin secretion and GABA_A_ receptor expression in rat pancreatic islets. (**A**) mRNA activity of GABA_A_ receptor subtypes α1, α2 and α3 in pancreatic islets is decreased after IH exposure (*n* = 3) compared with the control samples (*n* = 3). (**B**) Western blots showing the protein levels of corresponding GABA_A_ receptors, as indicated by arrows. Full-length blots are found in the Supplementary Information, Figure S2. (**C**) Serum insulin levels determined by ELISA 3 weeks after postnatal IH exposure compared with control animals. IH (open bar) group (*n* = 5); C (closed bar) Control group (*n* = 5). * indicates *p* = 0.0015, ** indicates *p* < 0.0001, *** indicates *p* = 0.0021.

The pups were euthanized at 3 weeks post-IH exposure. Compared with the controls, IH-treated animals exhibited significantly lower mRNA (Figure 4A) expression and corresponding protein levels of GABA_A_ α1, GABA_A_ α2 and GABA_A_ α3 receptors (Figure 4B). The serum insulin levels also decreased significantly, *p* < 0.0001 (Figure 4C).

### 3.4. Comparisons of Insulin Secretion Levels by Primary Islets Responding to Exogenous Addition of GABA and Bicuculine

Lastly, we examined the effects of exogenous 100 µM GABA (Cat. #0344, Tocris BioScience, Bristol, United Kingdom) and the effects of blocking GABA_A_ receptors by 100 µM bicuculine (Cat. #03131, Tocris BioScience, Bristol, United Kingdom) in both media with 2 mM and 16 mM glucose. One-way ANOVA tests with multiple comparisons were performed to compare the normal (baseline), GABA and Bic subgroups in 2 as well as 16 mM glucose.

**Figure 5:**
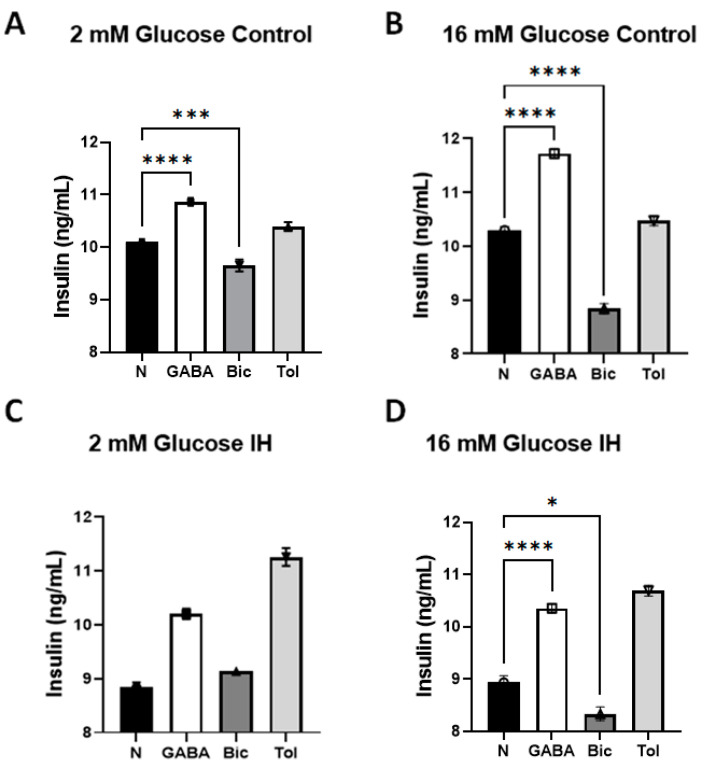
Changes in insulin secretion by primary islet cell groups (*n* = 3) responding to GABA and bicuculline (Bic) quantified by ELISA assays. (**A**) In 2 mM glucose media, the insulin secretion levels by the control islets increased with exogenous addition of GABA, and decreased with bicuculine. (**B**) In 16 mM glucose media, the insulin secretion levels by control islets increased with exogenous addition of GABA, and decreased with bicuculine. (**C**) No comparisons differed significantly, except for normal (N) versus 10 mM Tolbutamide (Tol) (*p* = 0.0448). (**D**) In 16 mM glucose medium for IH-treated islets, secretion increased with exogenous addition of GABA and decreased with bicuculine. * indicates *p* < 0.05, *** indicates *p* < 0.001 and **** indicates *p* < 0.0001. Comparisons between N vs. Tol were not indicated here; however, the addition of Tol elevated the insulin levels significantly at *p* < 0.05 when compared with each baseline N in each panel, except for (**B**) (*p* = 0.1294).

When GABA was added, insulin secretion by control islets elevated slightly in 2 mM glucose medium (10.10 ± 0.033 vs. 10.87 ± 0.042, *p* < 0.0001, see Figure 5A). When bicuculine was added to the control islets in 2 mM glucose, insulin secretion decreased from 10.10 ± 0.084 to 9.65 ± 0.066, *p* = 0.0009. With the addition of GABA, insulin secretion by the control islets in 16 mM glucose medium increased, as shown in Figure 4B (10.30 ± 0.042 vs. 11.73 ± 0.056, *p* < 0.0001). As shown in Figure 5C, IH-treated islets in 2 mM glucose produced no significant differences in pairs, except for N vs. Tol (*p* = 0.0448) after one-way ANOVA with Tukey’s multiple comparisons tests. Insulin secretion by IH islets tended to increase with GABA in 2 mM glucose medium (8.85 ± 0.049 vs. 10.20 ± 0.058, *p* = 0.3217), but the trend was not significant. When bicuculine was added to 2 mM glucose medium to IH islets, insulin secretion increased from 8.85 ± 0.048 to 9.14 ± 1.032, but these changes were also not significant due to increased variance (*p* = 0.9773).

Insulin secretion by IH islets in 16 mM glucose medium was elevated significantly by GABA, as shown in Figure 5D (8.94 ± 0.072 vs. 10.35 ± 0.052, *p* < 0.0001). In contrast, with bicuculine added to 16 mM glucose medium, insulin secretion by control islets was reduced markedly from 10.30 ± 0.042 to 8.84 ± 0.056, *p* = 0.0009 (See Figure 5B). With the IH-treated islets in 16 mM glucose, bicuculine decreased insulin secretion further, as shown in Figure 5D (8.94 ± 0.072 vs. 8.34 ± 0.075, *p* = 0.013). When tolbutamide was used for reference to evaluate the ATP-dependent potassium channel function of IH-treated islets in 2 mM or 16 mM glucose media, the insulin secretion levels increased significantly in each condition.

## 4. Discussion

Although the direction of cause–effect interactions between IH and T2D has not been clearly established, there is evidence that T2D can develop from IH insults, led by hypoxia-inducible factor (HIF)-1α pathways [20,21]. We investigated the potential role of GABA administration to T2D patients in cases with OSA in animal models.

The GABA levels in human plasma cover a wide range, yet they are known to be approximately 100–150 pmol/mL on average [22,23]. A previously reported clinical study using healthy volunteers showed that GABA significantly increases the circulating insulin levels in either fasting or fed conditions, with undisturbed insulin-to-glucagon ratios and glucose levels [8]. Pediatric patients with OSA who experience intermittent hypoxic breathing during sleep show increased levels of GABA in morning urine [24]. We still cannot confirm if this increased loss of GABA via morning urination in pediatric patients with OSA is associated with a significantly lower GABA level in the anterior insular cortices of OSA compared with healthy counterparts in adults [25]. We speculate that the altered anterior insular levels of GABA may modify integration and projections to autonomic areas in the human brain, possibly contributing to the impaired cardiovascular regulation in adults with OSA.

GABA is an excellent secretagogue of insulin [9,19,26]. Exogenous GABA increased the intracellular calcium ion levels, as shown in Figure 2, a finding consistent with previous reports [27,28]. Since an increased intracellular calcium ion level evokes insulin secretion [29], our data would also support the findings that GABA induces insulin secretion. The data from Figure 2 do not exhibit a cause–effect relationship between GABA-induced increases in intracellular calcium ion levels and ATP-induced intracellular calcium ion levels. Thus, we cannot assert that these two mechanisms occurred in a sequential fashion in pancreatic islets based on these findings. However, it is possible that GABA could couple with GABA_A_ receptors to evoke the depolarization of the β cells, and a subsequent activation of Ca^2+^ permeable ion channels, i.e., voltage-gated Ca^2+^ channels, may lead to an intracellular calcium increase, as previously suggested by others [28,30]. We noted that the calcium responses progressively declined in response to repeated GABA applications (Figure 2C). This outcome might result from the desensitization of GABA receptors [8,31] and/or inactivation of voltage-gated calcium ion channels [32]. However, the declining calcium responses to repeated GABA applications suggest a possibility that prolonged exposure of pancreatic beta cells to a high concentration (500 mM in our current experimental setup) of GABA could reduce the membrane depolarization levels, leading to gradual insulin secretion as GABA consumes GABA_A_ receptors. This process may underlie the mechanisms by which saturated GABA levels block insulin exocytosis [8].

Significant numbers of pediatric and adult patients with OSA are Type 2 diabetic or are prediabetic [2,33,34,35], with the extent of diabetic symptoms closely associated with the severity of OSA. The start/stop airflow pattern characterizing OSA results in IH. Repetitive IH conditioning induces a very rapid insulin decrease, as shown in Figure 3A. Our current (Figure 4C), and previous research [36] shows that such hypo-insulinemia is sustained for 3 weeks or longer subsequent to short-term IH exposure in rodents. The increasing prevalence of OSA and the increasing number of patients with T2D and prediabetes suggest that evaluating the therapeutic interaction of GABA with insulin-secretion processes under IH conditions is an urgent public health issue.

GABA has become a popular research target molecule for the remedial control of T2D. Exogenous supplementation of GABA appears to influence the insulin secretion levels under both control and IH-exposed conditions. Under fasting conditions, GABA elevated the insulin levels in normal breathing environments (See Figure 5A). After the islets were conditioned under IH, GABA actions could not significantly elevate insulin secretion in 2 mM glucose; however, in 16 mM glucose medium (assuming a postprandial-like condition), GABA elevated the insulin levels effectively, despite IH-conditioning. This outcome raises an important clinical conjecture that GABA could assist in insulin secretion after meals in prediabetic and diabetic patients with OSA. However, since GABA did not elevate the insulin levels in a fasting state, as shown in Figure 5C, further study is required to clarify this relationship. Follow-up studies are imperative to confirm the use of GABA during early morning fasting states, when OSA episodes are frequently observed.

We previously examined if neonatal IH exposure influences the mRNA levels of ATP-sensitive potassium (K_ATP_) channels, as indicated by the comparison of transcriptional activities of the associated genes (Sur1 and Kir6.2) between IH-treated and control islets. Conditioning with tolbutamide, a blocker of K_ATP_ channels, did not alter the KCC2 levels in response to IH, where the insulin levels increased in 2 mM glucose medium. However, the application of tolbutamide to control and IH-treated islets in 2 mM as well as 16 mM glucose media elevated the insulin secretion levels significantly. This outcome suggests that ATP-sensitive potassium channel-modulated insulin secretion appears to be an independent pathway from the GABA-dependent insulin secretion in the pancreas exposed to IH.

The current report lacks in vivo results of GABA’s effects. Although several clinical studies support our current results [37], we need to collect such translational data from rodents under tightly controlled experimental conditions to study GABA’s effects on insulin secretion in IH-conditioned animals under fasting and postprandial conditions. Nevertheless, we found that: (1) GABA–GABA_A_ receptor coupling action participates in the regulation of insulin secretion by pancreatic β cells in rat islets, and that influence is independent of the regulation of insulin exocytosis by ATP-dependent potassium channels; (2) IH challenges to rat islet cells decrease the levels of intracellular chloride via the increased chloride regressor KCC2 levels; (3) extracellular glucose levels did not influence insulin secretion as much as the effects of IH challenges; and (4) GABA-associated dysregulation of insulin secretion induced by IH exposure could be affected by the reduced concentration of GABA_A_ receptors.

We conclude that IH interferes with GABA action on insulin secretion. GABA may be an excellent insulin secretagogue in pediatric patients with OSA or periodic breathing if the concentration of functional GABA_A_ receptors is maintained. If GABA compensates for hypoinsulinemia during postprandial conditions in pediatric patients with hypopnea or periodic breathing, GABA could play a useful remedial role. This set of new information would be useful for studying the interactions of antidiabetic drugs and sleep, as discussed by others [13].

## Figures and Tables

**Table 1:** Sequences of gene-specific primers.

Gene	Sequence (5′ → 3′)
GABA_A_Rα1	F: CTCCTACAGCAACCAGCTATAC
*NM_183326*	R: GCTTGACTTCTTTCGGTTCTATG
GABA_A_Rα2	F: GACTCCTGACACCTTCTTTCAC
*NM_001135779*	R: CAGCAATGTTCCGTCATCCT
GABA_A_Rα3	F: GTCTCTCCAAGTTGCTGTCTAA
*NM_017069*	R: CCAGTGGTTCCAGGTAGAATAC
β-actin	F: ACAGGATGCAGAAGGAGATTAC
*NM_031144*	R: ACAGTGAGGCCAGGATAGA

## Data Availability

Data supporting reported results can be found with E.P.

## Supplementary Materials

The following supporting information can be downloaded at: https://www.mdpi.com/article/10.3390/children9091305/s1, Figure S1 and Figure S2.
