# Supplementary figures and images for: Intermittent Hypoxia Interferes with Autocrine Effects of GABA on Insulin Secretion in Postnatal Rodents—Implications for Pediatric Obstructive Sleep Apnea
Source: Children (Basel). 2022 Aug 28;9(9):1305. doi: 10.3390/children9091305 (PMC9497165; doi:10.3390/children9091305)

Supplementary Information Figure S1

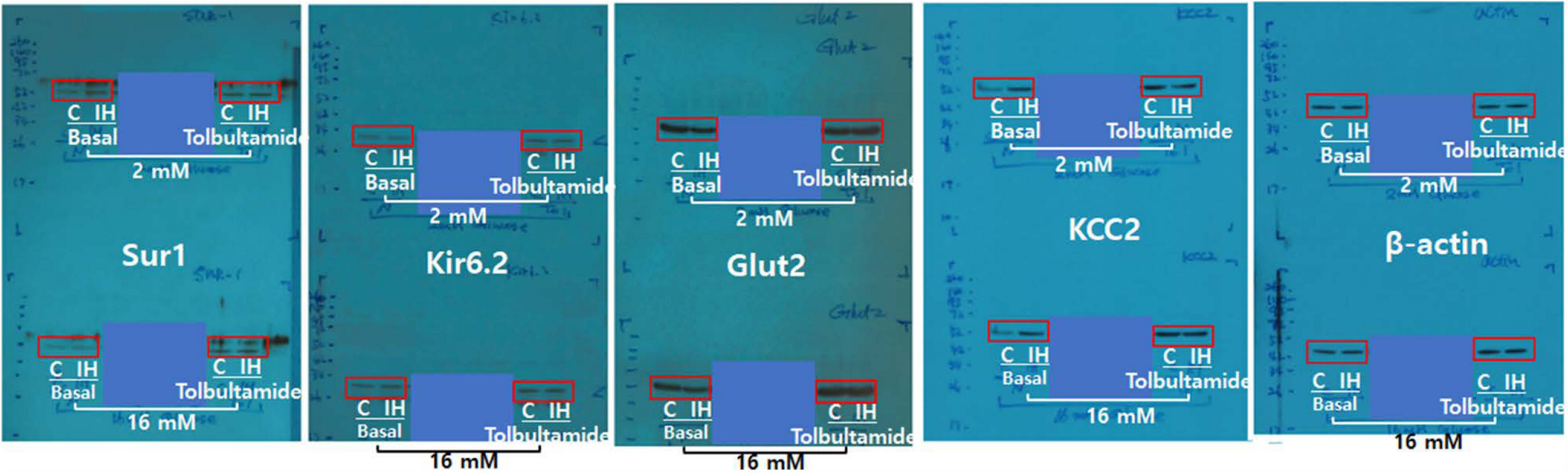

Supplementary Information Figure S2

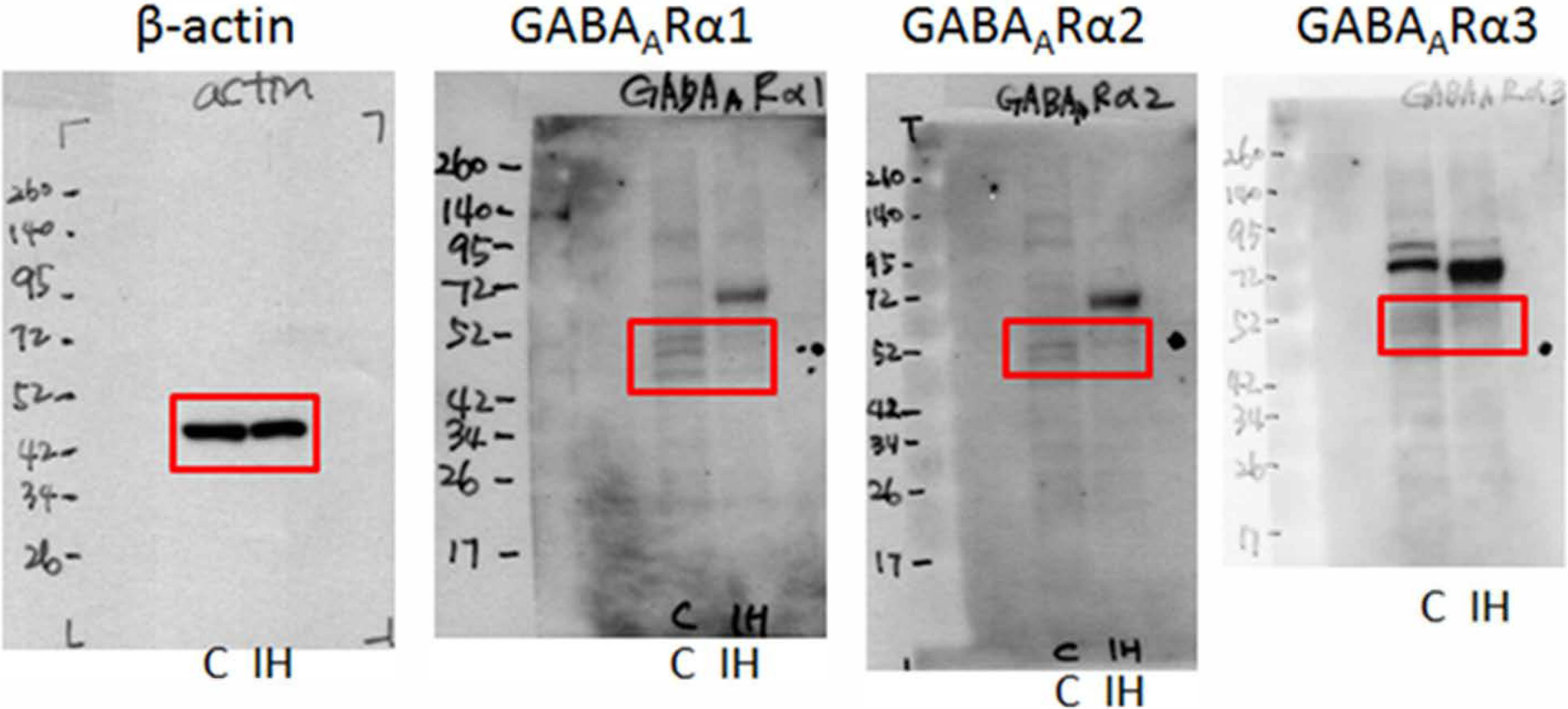

Supplement: Supplementary file 1 [file children-09-01305-s001.zip › children-1871192-supplementary.pdf]
